# Supplementary material for: Accurate plant pathogen effector protein classification ab initio with deepredeff: an ensemble of convolutional neural networks
Source: BMC Bioinformatics. 2021 Jul 17;22:372. doi: 10.1186/s12859-021-04293-3 (PMC8285798; doi:10.1186/s12859-021-04293-3)
Supplement: Supplementary file 1 — Additional file 1. Supplemental Tables. [file 12859_2021_4293_MOESM1_ESM.pdf]

Supplementary Tables

Table S1: Bacterial species contributing to the bacterial effector sequence set retrieved from PHI-Base

Species	Sequences Recovered
Clavibacter michiganensis	2
Cystobacter fuscus	1
Erwinia amylovora	10
Pantoea stewartii	2
Pseudomonas cichorii	1
Pseudomonas savastanoi	1
Pseudomonas syringae	72
Ralstonia solanacearum	46
Xanthomonas axonopodis	4
Xanthomonas campestris	19
Xanthomonas citri	5
Xanthomonas oryzae	24
Xylella fastidiosa	3

Table S2: Fungal species contributing to the fungal effector sequence set retrieved from PHI-Base

Species	Sequences Recovered
Blumeria graminis	14
Botrytis cinerea	1
Cercospora beticola	1
Colletotrichum orbiculare	1
Dothistroma septosporum	1
Fusarium graminearum	1
Fusarium oxysporum	9
Leptosphaeria maculans	6
Magnaporthe oryzae	19
Penicillium expansum	4
Puccinia striiformis	2
Pyrenophora tritici-repentis	2
Rhynchosporium commune	3
Ustilago maydis	25
Verticillium dahliae	6
Zymoseptoria tritici	2

Table S3: Oomycete species contributing to the oomycete effector sequence set retrieved from PHI-Base

Species	Sequences Recovered
Hyaloperonospora arabidopsidis	42
Phytophthora cactorum	1
Phytophthora infestans	20
Phytophthora parasitica	2
Phytophthora sojae	19
Pythium aphanidermatum	1

Table S4: List of possible combination of hyperparamaters setting for each model

Group	Model	Possible combination
Bacteria	CNN-LSTM	108
Bacteria	CNN-GRU	2304
Bacteria	LSTM Embedding	192
Bacteria	GRU Embedding	144
Fungi	CNN-LSTM	108
Fungi	CNN-GRU	1024
Fungi	LSTM Embedding	192
Fungi	GRU Embedding	216
Oomycete	CNN-LSTM	108
Oomycete	CNN-GRU	576
Oomycete	LSTM Embedding	128
Oomycete	GRU Embedding	2592

Table S5: Hyperparameters fixed in automatic scanning of hyperparameter space for each model

Group	Model	Fixed hyperparamaters	Values
Bacteria	CNN-LSTM	strides	1
Bacteria	CNN-LSTM	padding	valid
Bacteria	CNN-LSTM	activation_convolution	None
Bacteria	CNN-LSTM	activation_LSTM	tanh
Bacteria	CNN-LSTM	epochs	30
Bacteria	CNN-GRU	opt_go_backwards	TRUE
Bacteria	CNN-GRU	epochs	30
Bacteria	LSTM Embedding	opt_dropout_recurrent	0
Bacteria	LSTM Embedding	epochs	30
Bacteria	GRU Embedding	opt_dropout	0
Bacteria	GRU Embedding	opt_dropout_recurrent	0
Bacteria	GRU Embedding	epochs	30
Fungi	CNN-LSTM	strides	1
Fungi	CNN-LSTM	padding	valid
Fungi	CNN-LSTM	activation_convolution	None
Fungi	CNN-LSTM	activation_LSTM	tanh
Fungi	CNN-LSTM	epochs	30
Fungi	CNN-GRU	opt_go_backwards	TRUE
Fungi	CNN-GRU	epochs	30
Fungi	LSTM Embedding	opt_dropout_recurrent	0
Fungi	LSTM Embedding	epochs	30
Fungi	GRU Embedding	opt_dropout	0
Fungi	GRU Embedding	opt_dropout_recurrent	0
Fungi	GRU Embedding	epochs	30
Oomycete	CNN-LSTM	strides	1
Oomycete	CNN-LSTM	padding	valid
Oomycete	CNN-LSTM	activation_convolution	None
Oomycete	CNN-LSTM	activation_LSTM	tanh
Oomycete	CNN-LSTM	epochs	30
Oomycete	CNN-GRU	kernel_size	2
Oomycete	CNN-GRU	opt_go_backwards	TRUE
Oomycete	CNN-GRU	optimizers	Adam
Oomycete	CNN-GRU	epochs	30
Oomycete	LSTM Embedding	opt_dropout_recurrent	0
Oomycete	LSTM Embedding	epochs	30
Oomycete	GRU Embedding	epochs	30

Table S6: Hyperparameters tuned in manual scanning of hyperparameter space for each model

Group	Model	Tuned hyperpramaters	Values
Bacteria	CNN-LSTM	filters	4, 8, 16
Bacteria	CNN-LSTM	filters_LSTM	4, 8, 16
Bacteria	CNN-LSTM	optimizers	Adam, Adadelta
Bacteria	CNN-LSTM	number_hidden_units	4, 8
Bacteria	CNN-LSTM	batch_size	8, 16, 32
Bacteria	CNN-GRU	filter_conv	8, 16, 32
Bacteria	CNN-GRU	kernel_size	1, 2
Bacteria	CNN-GRU	maxpool_size	2, 3
Bacteria	CNN-GRU	activation_conv	None, relu
Bacteria	CNN-GRU	gru_hidden_units	8, 16, 32
Bacteria	CNN-GRU	opt_dropout	0, 0.25
Bacteria	CNN-GRU	opt_dropout_recurrent	0, 0.25
Bacteria	CNN-GRU	reg_rate	0.01, 0.001
Bacteria	CNN-GRU	optimizers	Adam, sgd
Bacteria	CNN-GRU	batch_size	8, 16
Bacteria	LSTM Embedding	outputdim	16, 32, 64
Bacteria	LSTM Embedding	lstm_hidden_units	16, 32
Bacteria	LSTM Embedding	opt_dropout	0, 0.25
Bacteria	LSTM Embedding	opt_go_backwards	TRUE, FALSE
Bacteria	LSTM Embedding	reg_rate	0.01, 0.001
Bacteria	LSTM Embedding	optimizers	Adam, sgd
Bacteria	LSTM Embedding	batch_size	8: 16
Bacteria	GRU Embedding	outputdim	32, 48
Bacteria	GRU Embedding	gru_hidden_units	8, 16, 32
Bacteria	GRU Embedding	opt_go_backwards	TRUE, FALSE
Bacteria	GRU Embedding	reg_rate	0.01, 0.001
Bacteria	GRU Embedding	optimizers	'sgd', 'Adam', 'Adadelta'
Bacteria	GRU Embedding	batch_size	16, 32
Fungi	CNN-LSTM	filters	4, 8, 16
Fungi	CNN-LSTM	filters_LSTM	4, 8, 16
Fungi	CNN-LSTM	optimizers	Adam, Adadelta
Fungi	CNN-LSTM	number_hidden_units	4, 8
Fungi	CNN-LSTM	batch_size	4, 8, 16
Fungi	CNN-GRU	filter_conv	8, 16
Fungi	CNN-GRU	kernel_size	1, 2
Fungi	CNN-GRU	maxpool_size	2, 3
Fungi	CNN-GRU	activation_conv	None, relu
Fungi	CNN-GRU	gru_hidden_units	8, 16
Fungi	CNN-GRU	opt_dropout	0, 0.25
Fungi	CNN-GRU	opt_dropout_recurrent	0, 0.25
Fungi	CNN-GRU	reg_rate	0.01, 0.001
Fungi	CNN-GRU	optimizers	Adam, sgd
Fungi	CNN-GRU	batch_size	4, 8
Fungi	LSTM Embedding	outputdim	16, 32, 64
Fungi	LSTM Embedding	lstm_hidden_units	16, 32
Fungi	LSTM Embedding	opt_dropout	0, 0.25
Fungi	LSTM Embedding	opt_go_backwards	TRUE, FALSE
Fungi	LSTM Embedding	reg_rate	0.01, 0.001
Fungi	LSTM Embedding	optimizers	Adam, sgd
Fungi	LSTM Embedding	batch_size	4, 8
Fungi	GRU Embedding	outputdim	8, 16, 32
Fungi	GRU Embedding	gru_hidden_units	8, 16, 32
Fungi	GRU Embedding	opt_go_backwards	TRUE, FALSE
Fungi	GRU Embedding	reg_rate	0.01, 0.001
Fungi	GRU Embedding	optimizers	'sgd', 'Adam', 'Adadelta'
Fungi	GRU Embedding	batch_size	4, 8
Oomycete	CNN-LSTM	filters	4, 8, 16
Oomycete	CNN-LSTM	filters_LSTM	4, 8, 16
Oomycete	CNN-LSTM	optimizers	Adam, Adadelta
Oomycete	CNN-LSTM	number_hidden_units	4, 8
Oomycete	CNN-LSTM	batch_size	4, 8, 16
Oomycete	CNN-GRU	filter_conv	8, 16, 32
Oomycete	CNN-GRU	maxpool_size	2, 3
Oomycete	CNN-GRU	activation_conv	None, relu
Oomycete	CNN-GRU	gru_hidden_units	8, 16, 32

Table S6: Hyperparameters tuned in manual scanning of hyperparameter space for each model *(continued)*

Group	Model	Tuned hyperpramaters	Values
Oomycete	CNN-GRU	opt_dropout	0, 0.25
Oomycete	CNN-GRU	opt_dropout_recurrent	0, 0.25
Oomycete	CNN-GRU	reg_rate	0.01, 0.001
Oomycete	CNN-GRU	batch_size	4, 8
Oomycete	LSTM Embedding	outputdim	16, 32, 64
Oomycete	LSTM Embedding	lstm_hidden_units	16, 32
Oomycete	LSTM Embedding	opt_dropout	0, 0.25
Oomycete	LSTM Embedding	opt_go_backwards	TRUE, FALSE
Oomycete	LSTM Embedding	reg_rate	0.01, 0.001
Oomycete	LSTM Embedding	optimizers	Adam, sgd
Oomycete	LSTM Embedding	batch_size	4, 8
Oomycete	GRU Embedding	outputdim	32, 48, 16
Oomycete	GRU Embedding	gru_hidden_units	8, 16, 32, 64
Oomycete	GRU Embedding	opt_dropout	0, 0.25, 0.5
Oomycete	GRU Embedding	opt_dropout_recurrent	0, 0.25, 0.5
Oomycete	GRU Embedding	opt_go_backwards	TRUE, FALSE
Oomycete	GRU Embedding	reg_rate	0.01, 0.001
Oomycete	GRU Embedding	optimizers	'sgd', 'Adam', 'Adadelta'
Oomycete	GRU Embedding	batch_size	4, 8

Table S7: Hyperparameters used in automatic scanning of hyperparameter space and best performing parameters values for each model

Group	Model	Paramater	BestValue
Bacteria	CNN-LSTM	strides	1
Bacteria	CNN-LSTM	padding	valid
Bacteria	CNN-LSTM	optimizers	Adadelta
Bacteria	CNN-LSTM	number_hidden_units	8
Bacteria	CNN-LSTM	filters_LSTM	16
Bacteria	CNN-LSTM	filters	16
Bacteria	CNN-LSTM	epochs	30
Bacteria	CNN-LSTM	batch_size	16
Bacteria	CNN-LSTM	activation_convolution	None
Bacteria	CNN-LSTM	activation_LSTM	tanh
Bacteria	CNN-GRU	reg_rate	0.001
Bacteria	CNN-GRU	optimizers	Adam
Bacteria	CNN-GRU	opt_go_backwards	TRUE
Bacteria	CNN-GRU	opt_dropout_recurrent	0
Bacteria	CNN-GRU	opt_dropout	0
Bacteria	CNN-GRU	maxpool_size	2
Bacteria	CNN-GRU	kernel_size	1
Bacteria	CNN-GRU	gru_hidden_units	16
Bacteria	CNN-GRU	filter_conv	16
Bacteria	CNN-GRU	epochs	30
Bacteria	CNN-GRU	batch_size	16
Bacteria	CNN-GRU	activation_conv	None
Bacteria	LSTM Embedding	reg_rate	0.001
Bacteria	LSTM Embedding	outputdim	16
Bacteria	LSTM Embedding	optimizers	Adam
Bacteria	LSTM Embedding	opt_go_backwards	TRUE
Bacteria	LSTM Embedding	opt_dropout_recurrent	0
Bacteria	LSTM Embedding	opt_dropout	0.25
Bacteria	LSTM Embedding	lstm_hidden_units	16
Bacteria	LSTM Embedding	epochs	30
Bacteria	LSTM Embedding	batch_size	16
Bacteria	GRU Embedding	reg_rate	0.001
Bacteria	GRU Embedding	outputdim	48
Bacteria	GRU Embedding	optimizers	Adam
Bacteria	GRU Embedding	opt_go_backwards	FALSE
Bacteria	GRU Embedding	opt_dropout_recurrent	0
Bacteria	GRU Embedding	opt_dropout	0

Table S7: Hyperparameters used in automatic scanning of hyperparameter space and best performing parameters values for each model *(continued)*

Group	Model	Paramater	BestValue
Bacteria	GRU Embedding	gru_hidden_units	16
Bacteria	GRU Embedding	epochs	30
Bacteria	GRU Embedding	batch_size	16
Fungi	CNN-LSTM	strides	1
Fungi	CNN-LSTM	padding	valid
Fungi	CNN-LSTM	optimizers	Adadelta
Fungi	CNN-LSTM	number_hidden_units	8
Fungi	CNN-LSTM	filters_LSTM	16
Fungi	CNN-LSTM	filters	16
Fungi	CNN-LSTM	epochs	30
Fungi	CNN-LSTM	batch_size	16
Fungi	CNN-LSTM	activation_convolution	None
Fungi	CNN-LSTM	activation_LSTM	tanh
Fungi	CNN-GRU	reg_rate	0.01
Fungi	CNN-GRU	optimizers	Adam
Fungi	CNN-GRU	opt_go_backwards	TRUE
Fungi	CNN-GRU	opt_dropout_recurrent	0
Fungi	CNN-GRU	opt_dropout	0
Fungi	CNN-GRU	maxpool_size	2
Fungi	CNN-GRU	kernel_size	1
Fungi	CNN-GRU	gru_hidden_units	8
Fungi	CNN-GRU	filter_conv	8
Fungi	CNN-GRU	epochs	60
Fungi	CNN-GRU	batch_size	8
Fungi	CNN-GRU	activation_conv	None
Fungi	LSTM-EMBEDDING	reg_rate	0.01
Fungi	LSTM-EMBEDDING	outputdim	16
Fungi	LSTM-EMBEDDING	optimizers	Adam
Fungi	LSTM-EMBEDDING	opt_go_backwards	FALSE
Fungi	LSTM-EMBEDDING	opt_dropout_recurrent	0
Fungi	LSTM-EMBEDDING	opt_dropout	0
Fungi	LSTM-EMBEDDING	lstm_hidden_units	32
Fungi	LSTM-EMBEDDING	epochs	60
Fungi	LSTM-EMBEDDING	batch_size	8
Fungi	GRU-Embedding	reg_rate	0.001
Fungi	GRU-Embedding	outputdim	16
Fungi	GRU-Embedding	optimizers	Adam
Fungi	GRU-Embedding	opt_go_backwards	FALSE
Fungi	GRU-Embedding	opt_dropout_recurrent	0
Fungi	GRU-Embedding	opt_dropout	0
Fungi	GRU-Embedding	gru_hidden_units	16
Fungi	GRU-Embedding	epochs	30
Fungi	GRU-Embedding	batch_size	8
Oomycete	CNN-LSTM	strides	1
Oomycete	CNN-LSTM	padding	valid
Oomycete	CNN-LSTM	optimizers	Adadelta
Oomycete	CNN-LSTM	number_hidden_units	4
Oomycete	CNN-LSTM	filters_LSTM	16
Oomycete	CNN-LSTM	filters	8
Oomycete	CNN-LSTM	epochs	30
Oomycete	CNN-LSTM	batch_size	16
Oomycete	CNN-LSTM	activation_convolution	None
Oomycete	CNN-LSTM	activation_LSTM	tanh
Oomycete	CNN-GRU	reg_rate	0.001
Oomycete	CNN-GRU	optimizers	Adam
Oomycete	CNN-GRU	opt_go_backwards	TRUE
Oomycete	CNN-GRU	opt_dropout_recurrent	0.25
Oomycete	CNN-GRU	opt_dropout	0
Oomycete	CNN-GRU	maxpool_size	2
Oomycete	CNN-GRU	kernel_size	2
Oomycete	CNN-GRU	gru_hidden_units	8
Oomycete	CNN-GRU	filter_conv	16
Oomycete	CNN-GRU	epochs	30

Table S7: Hyperparameters used in automatic scanning of hyperparameter space and best performing parameters values for each model *(continued)*

Group	Model	Paramater	BestValue
Oomycete	CNN-GRU	batch_size	8
Oomycete	CNN-GRU	activation_conv	relu
Oomycete	LSTM-Embedding	reg_rate	0.001
Oomycete	LSTM-Embedding	outputdim	64
Oomycete	LSTM-Embedding	optimizers	Adam
Oomycete	LSTM-Embedding	opt_go_backwards	FALSE
Oomycete	LSTM-Embedding	opt_dropout_recurrent	0
Oomycete	LSTM-Embedding	opt_dropout	0
Oomycete	LSTM-Embedding	lstm_hidden_units	32
Oomycete	LSTM-Embedding	epochs	30
Oomycete	LSTM-Embedding	batch_size	4
Oomycete	GRU-Embedding	reg_rate	0.01
Oomycete	GRU-Embedding	outputdim	32
Oomycete	GRU-Embedding	optimizers	sgd
Oomycete	GRU-Embedding	opt_go_backwards	TRUE
Oomycete	GRU-Embedding	opt_dropout_recurrent	0.25
Oomycete	GRU-Embedding	opt_dropout	0.25
Oomycete	GRU-Embedding	gru_hidden_units	16
Oomycete	GRU-Embedding	epochs	30
Oomycete	GRU-Embedding	batch_size	4
